# The Arterial Circle of the Brain in the Bawean Deer (*Axis kuhlii*)

**DOI:** 10.3390/ani14233410

**Published:** 2024-11-26

**Authors:** Maciej Zdun, Jakub Jędrzej Ruszkowski, Maria Nabzdyk, Aleksander F. Butkiewicz, Maciej Gogulski, Marcin Gołyński

**Affiliations:** 1 Department of Basic and Preclinical Sciences, Nicolaus Copernicus University in Torun, Lwowska 1, 87-100 Torun, Poland; maciej.zdun@up.poznan.pl (M.Z.); butkiewicz@umk.pl (A.F.B.); 2 Department of Animal Anatomy, Poznan University of Life Sciences, Wojska Polskiego 71C, 60-625 Poznan, Poland; maria.nabzdyk@up.poznan.pl; 3 Department of Preclinical Sciences and Infectious Diseases, Poznan University of Life Sciences, Wołynska 35, 60-637 Poznan, Poland; maciej.gogulski@up.poznan.pl; 4 Veterinary Medicine Institute, Nicolaus Copernicus University in Torun, Lwowska 1, 87-100 Torun, Poland; marcingolynski@umk.pl

**Keywords:** anatomy, conservation, neuroanatomy, vascularization

## Abstract

The Bawean deer (*Axis kuhlii*) is a small deer species endemic to the island of Bawean in Indonesia. The species is listed as critically endangered by the IUCN Red List. The current population is assessed to be less than 500 adults living in the wild. The cerebral arterial circle (also called the circle of Willis) is an anastomosis of arteries that supply the brain. The aim of this study was to describe the arterial vascularization of the brain in this species. Three different methods were used to obtain a complete arterial pattern of this region—latex injection, corrosion cast, and contrast-enhanced computed tomography. The arterial vascularization of the brain was described. The pattern of the arterial vessels supplying the brain in this species was similar to that described previously by ruminants. The vessel with the biggest lumen branching off from the circle of Willis was the medial cerebral artery. The basilar artery is a vessel with a narrow lumen and does not mediate the delivery of blood to the encephalon from the caudal side. The results of the study may be useful in creating veterinary protocols for treating vascular diseases in this species and further studies from the field of pathophysiology or pathology.

## Simple Summary

The Bawean deer (*Axis kuhlii*) is a critically endangered small deer species endemic to the island of Bawean in Indonesia. This study aimed to describe the arterial blood supply to the brain in this species using three different angiological methods. This is the first description of this anatomical area in this species of deer.

## 1. Introduction

The Bawean deer (*Axis kuhlii*), also known as Kuhl’s hog deer or Bawean hog deer, is a deer species belonging to the genus Axis, endemic to the island of Bawean in Indonesia. The species is listed as critically endangered by the IUCN Red List. Among the threats are loss of environment, road accidents, and hunting are listed [1]. The current population is stable and is assessed to be 200–500 adults living in the wild [2]. In addition, this species is kept in zoos around the world.

The cerebral arterial circle (also called the circle of Willis [3,4,5]) is an anastomosis of arteries that supply the brain. It is formed by the junction of the bilateral rostral cerebral artery and the bilateral caudal communicating artery with the basilar artery caudally [6]. It provides a collateral blood flow between the anterior and posterior arterial systems of the brain. The circle of Willis is located at the base of the brain. Depending on the species, some differences in the components of this structure may occur with varying frequency [7,8]. In humans, these differences are clinically significant, predisposing to transient ischemic attack and stroke [9].

To date, no work has been published on the vascular anatomy of this species. Describing the detailed vascularization of individual body structures may contribute to expanding knowledge of the anatomy and physiology of the species. Moreover, it may constitute the basis for research on the pathophysiology and veterinary care of the species, which may provide indirect assistance in the conservation of this species of deer.

Understanding the detailed vascular anatomy of the structures supplying blood to the brain may provide an excellent basis for research on vascular diseases and their complications. In the course of these diseases, thromboembolic events may occur, affecting the cerebral vascular system [10]. This knowledge may also be important in cases of congestion and syncope because the brain is extremely prone to hypoxia and cannot store glucose and glycogen [11,12].

Due to the fact that the described species is rare and belongs to a group of species of special concern in the context of species protection, learning the basics of its body structure may be an excellent basis for the development of research on treatment protocols for veterinarians.

In this article, the anatomy of the arterial vessels of the brain in Bawean deer was described for the first time. This study aimed to describe the arterial vascularization of the brain in Bawean deer.

## 2. Materials and Methods

### 2.1. Animals

The research was performed on four adult (two males and two females) Bawean deer (*Axis kuhlii*) skulls. A post-mortem material has been delivered from Polish zoological gardens. The animals were under continuous veterinary care while alive. All individuals included in this study had been euthanized with intramuscular xylazine 10 mg/kg and ketamine 90 mg/kg, followed by intravenous pentobarbital 100 mg/kg for medical reasons other than for neurological, cardiac, or vascular diseases. None of the animals were euthanized for the cause of this experiment. Animals with trauma to the head or neck region have not been included in the study. All cadavers were frozen after death and thawed before the anatomical analysis.

### 2.2. Methods

The analysis was based on three different methods: corrosion-cast specimens, latex-injected specimens, and contrast-enhanced CBCT studies. The specimens have been randomly selected to be used in one of the methods. Two individuals have bilateral common carotid arteries injected with a red-colored solution of the chemo-setting acrylic material Duracryl^®^ Plus (SpofaDental, Jičín, Czech Republic) was used. When the substance became hard, the specimens were submerged in hot water with a detergent (Persil^®^, Düsseldorf, Germany) for an enzymatic maceration process in a special maceration tank. The water temperature was set to 40 °C, and the process lasted 37 days. The final result of the process was a cast of arterial blood vessels on the skull.

One specimen was used for another method, which consisted of injecting LBS 3060 latex (Synthos, Oświęcim, Poland) in a liquid form into the bilateral common carotid arteries. Specimens were cured in 5% formaldehyde and rinsed with water for forty-eight hours after that to get rid of excess for the main solution. The ventilation system was used for additional protection. It was set for 15 air changes per hour. The preparation of the soft tissue was enabled by cutting a square-shaped window in the skull bone with an oscillating saw. Afterward, the arteries were prepared by hand using preparation instruments. The anatomical examination started with removing the skin from the entire neck and head. The muscle tissue was then carefully dissected, taking care not to damage the surrounding soft tissues, including blood vessels. The next step was to clean the vessels of excess connective tissue. In this way, the preparations of arterial vessels, which were arranged physiologically on the surrounding soft tissues, were created.

Potential limitations of the experiment include problems with the density of both the latex and acrylic media used. The increased density prevents these agents from reaching narrow, distally located arteries. However, thanks to the use of material of this density, the preparations are more durable and less susceptible to damage, and it is possible to visualize the vessels clearly. Another limitation of those methods and the contrast-enhanced CT method is the possibility of leakage of the contrast agent through damaged vessels; however, in the case of this study, this was not a problem.

One cadaver was used for contrast-enhanced CT examination. Prior to the scans, the bilateral common carotid arteries were injected with a contrast agent (barium sulfuricum 1.0 g/mL, Medana, Sieradz, Poland). Cone-beam computed tomography (Fidex Animage, Pleasanton, CA, USA) was performed at the University Centre for Veterinary Medicine in Poznan, Poland. Parameters used for the study were 115 kVp, 0.08 mAs pet shot, 20.5 mAs (Total mAs), and a reconstructed slice thickness of 0.25 mm. Subsequently, the scan was reconstructed with the maximum intensity projection.

The nomenclature of the described structures is based on Nomina Anatomica Veterinaria and other papers on this topic [3,4,5,13].

The material was photographed and subjected to descriptive analysis. The results of the observations were compared with other groups of mammals. The photos were taken using a digital camera (Nikon D3200, Tokio, Japan). The photographs were saved in JPG format. GIMP v2.10.18, digital image editing software, was used to process the photographs.

## 3. Results

**Figure 1:**
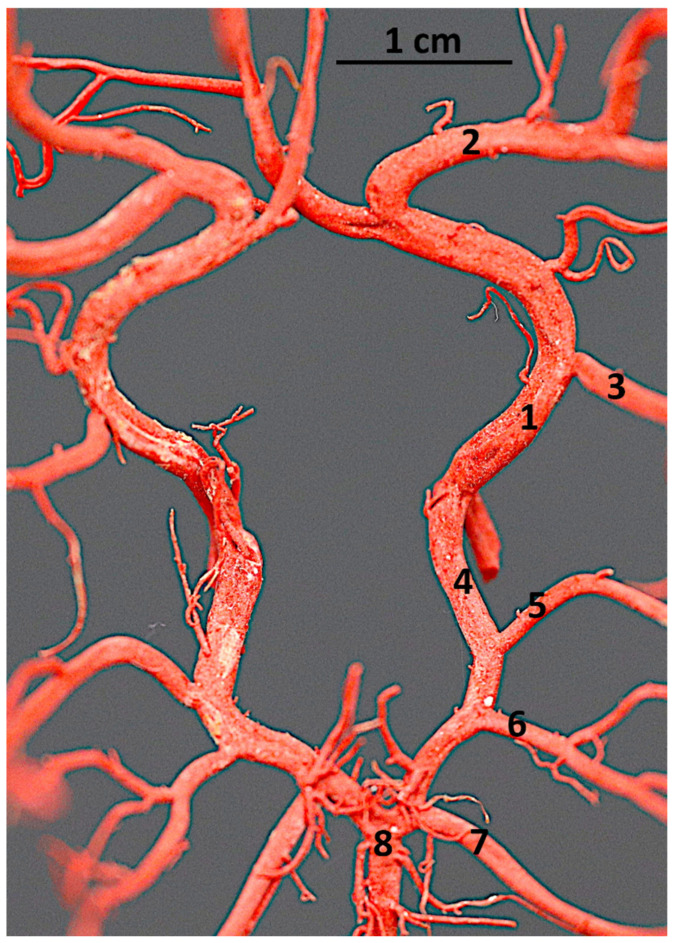
Isolated fragment of the corrosion cast preparation of the cerebral arterial circle of the Bawean deer. Dorsal view. 1—the rostral cerebral artery; 2—the middle cerebral artery; 3—the rostral choroidal artery; 4—the caudal communicating artery; 5—the caudal cerebral artery; 6 and 7—the rostral cerebellar artery; 8—the basilar artery.

The arterial circle of the brain (*circulus arteriosus cerebri*; circle of Willis) is created by bilateral rostral cerebral arteries (*arteriae cerebri rostrales*) and caudal communicating arteries (*arteriae communicans caudales*). These vessels are formed as a result of the division of the intracranial part of the internal carotid artery (*arteria carotis interna*) on each side of the body. The shape of the circle can be likened to a heart. Its rostral part is a little bit wider (Figure 1).

**Figure 2:**
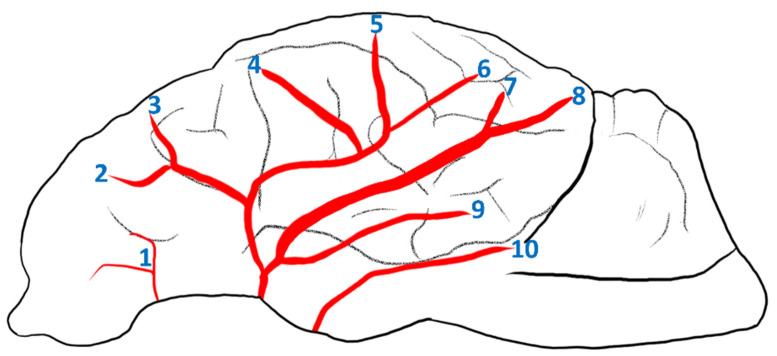
Scheme showing branches on the lateral surface of the brain. 1—rostral olfactory artery; 2—orbital branch; 3—inferior frontal branch; 4—superior frontal branch; 5—rostral parietal branch; 6—caudal parietal branch; 7—superior temporal branch; 8—middle temporal branch; 9—inferior temporal branch; 10—caudal olfactory artery.

The rostral cerebral arteries that form this part of the arterial circle of the brain are arranged in the shape of the letter C in the initial section of this vessel. About halfway through this length, the rostral choroidal artery (*arteria choroidea rostralis*) branches off. Near the end of the first section of the rostral cerebral artery, the middle cerebral artery (*arteria cerebri media*) branches off. In one case, unilaterally, this vessel branches off by two branches that create a vascular loop and then join together. This vessel lies on the lateral surface of the brain. Its main trunk creates 2–3 vessels that then branch into smaller vessels supplying the lobes of the brain (Figure 2).

Into the rostral direction, the orbital and inferior frontal branches are heading. Both branch off from a common trunk. The middle part of the brain is supplied by the superior frontal branch, the rostral parietal branch, and the caudal parietal branch. These vessels are located in the area of the middle suprasylvian sulcus. The caudal part of the brain is supplied by the temporal branches. The superior temporal, the middle temporal, and the inferior temporal branches are noted. The first two branch off with a common trunk. The superior temporal branch runs in the direction of the caudal suprasylvian sulcus and next in the direction of the ectomarginal sulcus. The middle temporal branch runs to the caudal suprasylvian sulcus. The inferior temporal branch branches off independently from the middle cerebral artery. The middle temporal branch and the inferior temporal branch supply the occipital lobe. More ventrally, the caudal olfactory artery is present. This vessel branches off from the rostral cerebral artery.

When the middle cerebral artery branches off, the rostral cerebral artery changes the direction of the course. This second section lies in the longitudinal fissure and supplies the rostral and middle parts of the encephalon from its medial surface.

**Figure 3:**
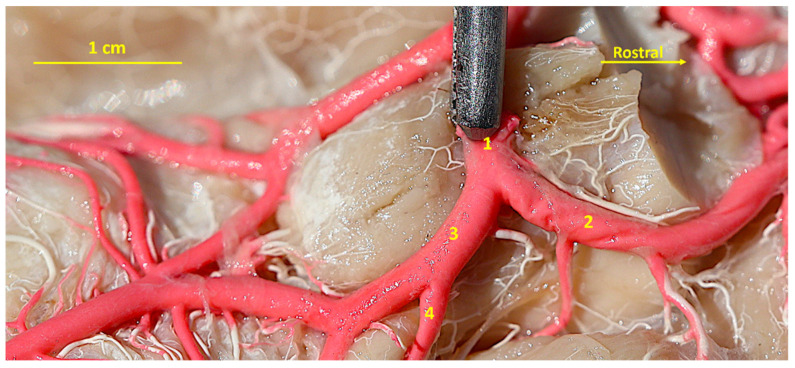
A latex specimen of the cerebral arterial circle of the Bawean deer. Ventro-lateral view. 1—the intracranial part of the internal carotid artery; 2—the rostral cerebral artery; 3—the caudal communicating artery; 4—the caudal choroidal artery.

Moreover, at the beginning of the second section, two more vessels branch off. One of them is the rostral communicating artery (*arteria communicans rostralis*). This small vessel anastomoses with a single-name vessel on the other side. The second vessel is the internal ethmoidal artery (*arteria ethmoidalis interna*). This is a strong vessel that lies on the medial surface of the olfactory bulb. Before this artery reaches the ethmoid bone, the vessels to the ventromedial surface of the rhinencephalon branch off as the rostral olfactory artery (Figure 3).

The caudal part of the circle of Willis is created by the bilateral caudal communicating arteries. These vessels are characterized by an arched puncture, which is not as strongly curved as the rostral cerebral arteries. From this artery, the caudal choroidal artery (*arteria choroidea caudalis*) branches off.

**Figure 4:**
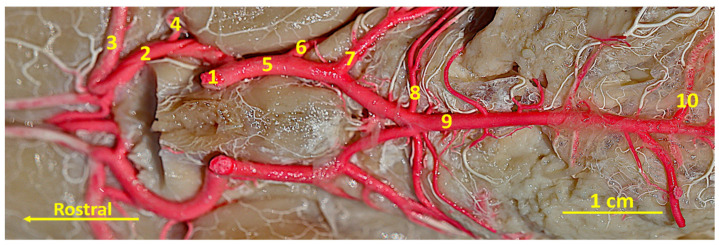
A latex specimen of the cerebral arterial circle of the Bawean deer. Ventral view. 1—the intracranial part of the internal carotid artery; 2—the rostral cerebral artery; 3—the middle cerebral artery; 4—the rostral choroidal artery; 5—the caudal communicating artery; 6—the caudal cerebral artery; 7 and 8—the rostral cerebellar artery; 9—the basilar artery; 10—the caudal cerebellar artery.

Next, the caudal cerebral artery (*arteria cerebri caudalis*) branches off. This single vessel supplies the caudal part of the brain from the caudal and ventral sides. More caudally, two rostral cerebellar arteries (*arteriae cerebelli rostrales*) branch off (Figure 4). One of them branches off close to the caudal cerebral artery. This one is a vessel with a wider lumen. The second one, with a smaller lumen, branches off more caudally at the point where the caudal communicating arteries evolve into the basilar artery (*arteria basilaris*).

Both rostral cerebellar arteries supply the main part of the cerebellum from the rostral, dorsal, and lateral sides.

**Figure 5:**
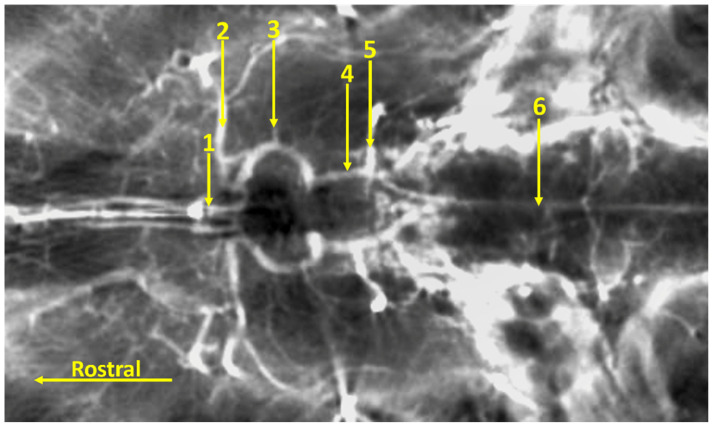
Maximum intensity projection reconstruction of the CT scan of the arterial circle of the brain and its branches in the Bawean deer. 1—the rostral cerebral artery; 2—the middle cerebral artery; 3—the rostral choroidal artery; 4—the caudal communicating artery; 5—the caudal cerebral artery; 6—the basilar artery.

The basilar artery is the odd artery that heads caudally. Its connection to the caudal communicating arteries contributes to closing the circle of Willis from the caudal side. On the caudal side, this vessel becomes narrow in comparison to its rostral part (Figure 4 and Figure 5). This vessel branches off the caudal cerebellar artery (*arteria cerebelli caudalis*), which complements the vascularization of the cerebellum.

## 4. Discussion

The circle of Willis in the Bawean deer has many features in common with other described species of cervids or, more broadly, ruminants. The presence of the rostral communicating artery was described. This vessel was described in all presented reindeer, chital, Eld’s deer, wapiti, sika deer, fallow deer, Pere David’s deer, Chinese muntjac, Eurasian elk, and yak [14,15,16]. Asymmetry of branching off this vessel was described in Eurasian elk and roe deer. In vascular variants, the rostral communicating artery branches off unilaterally or bilaterally from the rostral cerebral artery even before the point of branching off the middle cerebral artery [15,17]. It was double in some representatives of sheep [18]. The rostral communicating artery formed a network of small vessels in most specimens of the buffalo and in some specimens of Eurasian elk, roe deer, goat, and sheep [6,15,17,18,19]. In a study by König [20] in domestic cattle, this artery was absent in most cases. This causes the circle of Willis in such individuals to be open from the rostral side. Different observations were described by Brudnicki and Gielecki [21]. They state that this artery was present in almost 90% of examined cattle specimens. The rostral communicating artery was inconstant in cattle, and there was a lack of this artery in about 15% of sheep and goats and 2% of buffalo [5,7,18,19].

The vessel with the widest lumen that branched off from the circle of Willis is the middle cerebral artery. Single cases have been described in Eurasian elk and roe deer, in which this vessel was double [15,17]. Numerous works present the course of this vessel in selected representatives of cervids. The observation made in the Bawean deer is confirmed in other species in this group of animals, such as fallow deer, roe deer, or Eurasian elk [22,23,24]. In addition, studies regarding buffalo, cattle, and sheep also indicate the presence of branches with an arrangement like that of the Bawean deer [19,25,26]. Additionally, in a buffalo, a small branch from the rostral cerebral artery to the olfactory bulb (rostral olfactory artery) was noted [19].

The rostral choroidal artery branched off from the rostral cerebral artery. However, individual cases have been described in Eurasian elk, in which this vessel branched off the caudal communicating artery. This vessel has also been found to occur in a double form in this species, like in the roe deer [15,17]. In yak and some individuals of reindeer, cattle, and roe deer, this vessel branches off more caudally than in others, i.e., near the point where the rostral cerebral artery evolves from the intracranial part of the internal carotid artery, from the intracranial part of the internal carotid artery or even from the caudal communicating artery [14,17,21,27].

The caudal cerebral artery in double form was described in one-third of specimens of the Eurasian elk [15]. In addition, in Eurasian elk, triple caudal cerebral arteries were observed in some individuals. In the roe deer, the presence of this vessel in a double form was a feature of the vascular pattern [28]. However, Jabłoński describes this artery as a single vessel [17]. The caudal choroidal artery, as a branch of the mesencephalic artery, was noted in a yak [27]. In animals with multiple rostral cerebellar arteries, one branch departs from the basilar artery and one from the caudal communicating artery. This manner of branching off the rostral cerebellar artery was found in some representatives of the buffalo, Sri Lankan spotted chevrotain, goat, 10% of the roe deer, and more than one-third of Eurasian elk specimens [6,15,17,19,29].

The basilar artery is weaker from its caudal side. This observation was made in different ruminants, such as reindeer, chital, Eld’s deer, wapiti, sika deer, fallow deer, red deer, roe deer, Pere David’s deer, Chinese muntjac, and Eurasian elk, as well as in representatives of the Antilopinae subfamily, Bovini tribe, goats, giraffe, nilgau, niala, and eland [6,28,30,31,32,33,34,35,36]. Baldwin and Bell [37,38,39] conducted a series of physiological experiments on sheep and cattle, during which attention was paid to the direction of blood flow through this vessel. The results of the study showed that the basilar artery does not mediate the delivery of blood to the encephalon from the caudal side. They found only blood flow from the rostral side, i.e., from the caudal communicating arteries, into the caudal direction.

The caudal cerebellar artery in double form was noted in some individuals of the buffalo and a goat [6,19].

## 5. Conclusions

The article presents information on the detailed supply of arterial blood to the brain in the Bawean deer. The use of three anatomical methods, including modern imaging techniques, enabled the visualization of the described structures. The information contained in this article may constitute the basis for further research in the fields of physiology, pathophysiology, and diseases of this species.

## Data Availability

The data presented in this study are available on request from the corresponding author.
